# Nanotechnology-Based Approach in Tuberculosis Treatment

**DOI:** 10.1155/2017/4920209

**Published:** 2017-01-22

**Authors:** Mohammad Nasiruddin, Md. Kausar Neyaz, Shilpi Das

**Affiliations:** ^1^Triesta Sciences, HealthCare Global Enterprises Limited, Bangalore 560 027, India; ^2^Department of Research and Education, Artemis Hospitals, Sector 51, Gurgaon 122 001, India

## Abstract

Tuberculosis, commonly known as TB, is the second most fatal infectious disease after AIDS, caused by bacterium called* Mycobacterium tuberculosis*. Prolonged treatment, high pill burden, low compliance, and stiff administration schedules are factors that are responsible for emergence of MDR and XDR cases of tuberculosis. Till date, only BCG vaccine is available which is ineffective against adult pulmonary TB, which is the most common form of disease. Various unique antibodies have been developed to overcome drug resistance, reduce the treatment regimen, and elevate the compliance to treatment. Therefore, we need an effective and robust system to subdue technological drawbacks and improve the effectiveness of therapeutic drugs which still remains a major challenge for pharmaceutical technology. Nanoparticle-based ideology has shown convincing treatment and promising outcomes for chronic infectious diseases. Different types of nanocarriers have been evaluated as promising drug delivery systems for various administration routes. Controlled and sustained release of drugs is one of the advantages of nanoparticle-based antituberculosis drugs over free drug. It also reduces the dosage frequency and resolves the difficulty of low poor compliance. This paper reviews various nanotechnology-based therapies which can be used for the treatment of TB.

## 1. Introduction

Despite the remarkable advancement in medical science and therapeutics, tuberculosis (TB) still remains the primary factor of mortality and socioeconomic disaster for millions of people around the world. It has plagued humankind throughout known history and human prehistory. Tuberculosis is a deadly infectious disease caused by the bacteria* Mycobacterium tuberculosis* [1]. Robert Koch in 1882 was awarded Nobel Prize for this remarkable discovery.* Mycobacterium tuberculosis* (Mtb) is an acid-fast bacillus and intracellular pathogen, which has developed numerous strategies to avoid being killed by macrophages [2, 3]. It is considered as the most successful pathogen capable of persisting in host for decades without causing the disease [4]. Tuberculosis is one of the main causes of mortality and morbidity globally. According to World Health Organization (WHO),* M. tuberculosis* (Mtb) has infected approximately one-third of the world's population, effecting more than 9 million new cases and 2 million deaths annually, and the rest of the infected people remain asymptomatic [5]. Tuberculosis causes more deaths each year than any other infectious disease and is second after AIDS among infectious diseases [6]. Therefore, in 1993, WHO declared tuberculosis a global health emergency [7].* Mycobacterium* primarily attacks the lungs, but it can attack any part of the body, like the kidney, lymphatic system, central nervous system (meningitis), circulatory system (miliary tuberculosis), genitourinary system, joints, and bones [8]. The success and failure rate to treat tuberculosis depends on various factors like (a) patient compliance to the treatment taken, (b) malnutrition, (c) smoking, (d) coexisting diseases like HIV, and (e) inadequate supervision by health care staffs.

The critical problem with the current tuberculosis chemotherapy is that when the drug is taken intravenously or administered orally, it is distributed throughout the body via the systemic blood circulation and a majority of molecules do not reach their targets and, consequently, stay in the body causing adverse side effects. Drugs have a short plasma-life and rapid clearance, which limits their effectiveness [9]. To defeat the challenges posed by antituberculosis drugs and to recover the accomplishment rate of tuberculosis treatment, we need to have new tuberculosis drugs (Table 1), which can overcome these challenges.

The clinical management of tuberculosis still remains a difficult task. A large number of tuberculosis endemic areas are being covered for antitubercular treatment (ATT) under directly observed treatment. Short course (DOTS) program of World Health Organization (WHO) has not been completely successful in controlling TB and major burden in developing countries of Indian subcontinent and Africa [10].

The major problems associated with treatment of TB are long duration of treatment and continuous and frequent multiple drugs dosing which leads to low or noncompliance of patients to current therapy. Low compliance of patients is the main contributory factor in the reemergence of the disease and the development of multidrug-resistant (MDR) tuberculosis and more severe form called extensively drug-resistant (XDR) tuberculosis [11]. Moreover, the prevalence of multidrug-resistant tuberculosis (MDR-TB and XDR-TB) is increasing in developing countries. That causes a challenge to the medical sciences and became matter of great concern. AIDS is again a major contributor to the increasing incidence of tuberculosis, providing fertile grounds for Mtb to grow in an immunocompromised host [12]. The alarm of spread of the MDR and XDR strains and the lack of successful treatment options strengthen the need to develop new and effective anti-TB drugs to overcome the problem of drug resistance, shorten the treatment course, and better compliance [13].

## 2. Different Forms of TB

There are two forms of TB, latent TB and active TB. In latent TB, bacteria remain dormant in body. This phase can last for much longer time. It is usually treated by taking one medicine for 9 months. And, in the active TB, bacteria multiply and spread in the body, thereby causing damage to the tissue [8].

## 3. Multidrug-Resistant Tuberculosis (MDR-TB)

This is onerous form of tuberculosis (TB) defined by resistance to at least two of the standard four drug anti-TB medicines (first-line antituberculosis drugs) [23]. Inadequate or inconsistent treatment has allowed MDR-TB to emerge and spread quickly. Today, the treatment for drug-resistant TB takes almost two years and, in addition, the treatment is so complex, expensive, and toxic that MDR-TB patients struggle to live [24]. Treatment of MDR-TB consists of second-line drugs. Many second-line drugs are lethal and have harsh side effects. Treatment for MDR-TB is administered for 2 years or more than that and involves daily injections. All these components hold a significant challenge to governments and health care departments. World Health Organization aims to treat 80% of the MDR-TB cases by 2015. Without unique, simple, and inexpensive treatments for MDR-TB, this is next to impossible. WHO predicted that more than 2 million people would have developed MDR-TB between 2011 and 2015 [25].

## 4. Extensively Drug-Resistant TB (XDR-TB)

XDR-TB poses a major risk to public health. This is more brutal form of MDR-TB and is characterized by resistance to any fluoroquinolone and at least one of the three injectable second-line drugs [26]. This makes this XDR-TB treatment extremely problematic. In the year 2006, XDR-TB outbroke in KwaZulu-Natal, South Africa; 52 out of 53 people who contracted the disease died within few months [24]. 70% of XDR-TB patients were estimated to die within a month of diagnosis. Estimation by WHO suggested that roughly 5% of MRD-TB cases are XDR-TB.

## 5. Drug Regimens

### 5.1. First-Line Drugs

In general, tuberculosis is treated with first-line drugs as a combination therapy with isoniazid, rifampin, pyrazinamide, and ethambutol for several months. These drugs are administered orally and have outstanding effectiveness against Mtb [27].

### 5.2. Second-Line Drugs

When Mtb strain is resistant to isoniazid and rifampin, two of the most powerful first-line drugs, it develops into more complex form of TB known as MDR-TB. A combination of second-line drugs used to cure MDR-TB is aminoglycosides such as amikacin and kanamycin, polypeptides such as capreomycin, viomycin, and enviomycin, fluoroquinolones such as ciprofloxacin, levofloxacin, and moxifloxacin, and thioamides such as ethionamide, prothionamide, and cycloserine [27]. Second-line drugs are more lethal and are more expensive than first-line drugs, and treatment may last much longer [8].

### 5.3. Third-Line Drugs

The third-line drugs for treating TB include rifabutin, linezolid, thioridazine, arginine, vitamin D, and macrolides such as clarithromycin and thioacetazone [27]. Like other drugs for the treatment of TB, third-line drugs are not as effective or their efficacy has not been proven [28]. Third-line drugs are also not listed by WHO. New advanced technologies like the design of carrier-based drug delivery system are under the inspection for treatment of TB. Biodegradable polymers, liposomes, and microsphere were developed in order to reduce the dose and duration of treatment [29]. The drugs are gradually released with high concentration and minimum toxicity as compared to the commonly used drugs.

Although current anti-TB drugs are effective, urgent strategies must be developed in order to accomplish delivery of these drugs. In this context, nanotechnology is one of the most promising passages for development of more decisive and more effective drug delivery systems for treatment of TB along with the potent strategy for the development and delivery of next-generation TB vaccines.

Nanotechnology-based drug delivery system has ability to improve tolerability of noxious chemotherapies, sustained and controlled drug release, and eventually increased bioavailability. Desired particle size required for drug localization upon administration by inhalation is between 50 and 200 nm.

Phagocytic escape by slow release from lungs and mucociliary clearance without inducing any immune responses are promising key factors of nanosized particles. Rapid drug absorption through the pulmonary epithelia and high lung bioavailability facilitate the lowering of drug doses and also maintaining therapeutic concentration. Combination of smaller dose with absence of first pass metabolism and prevention of gastrointestinal tract is estimated to reduce to systemic side effects and increased tolerability. The novel design of anti-TB antibiotics, which is currently followed to deal with the resistant strains of mycobacterium, is designed as such to shorten the treatment course and limit drug communications with other anti-TB and anti-HIV drugs [30]. If managed properly, first-generation anti-TB drugs can still show better efficacy. In this context, nanotechnology has emerged as a promising area to rise above the limitations of some of the following factors: (a) increased patient conformity and devotion to regimes, (b) main technological margins, and (c) targeting bacterial reservoirs (e.g., alveolar macrophages) [31].

## 6. Pathogenesis and Immunology of TB

The first stage of tuberculosis is initiated with inhalation of droplets generated by a person with active tuberculosis. These droplets can remain for a longer time in the air. When inhaled, a single droplet may be enough to cause the disease. Most droplets end up in the upper respiratory tract, where the microbes are killed, but a few penetrate further down. The bacteria reach the alveoli in the lungs, where the alveolar macrophages phagocytose them. Several receptors are involved in the uptake process including mannose receptors, Toll-like receptor 2 (TLR2) and Toll-like receptor 4 (TLR4), surfactant protein A receptors, CD14, scavenger receptors, complement receptors, and immunoglobulin receptors [32]. Sometimes macrophages fail to destroy the bacteria either because compounds produced by the microbe inactivate them or because phagosome-lysosome fusion mechanisms are inhibited by* M. tuberculosis*, thereby avoiding low pH exposure and hydrolytic surroundings of phagolysosomes [33].

In the second stage, mycobacterium multiplies in the macrophage, eventually causing its lysis. This results in the cellular damage which attracts the inflammatory cells and blood monocytes to the area. Monocytes differentiate into macrophages and attempt to attack the microbe which is ingested by the macrophages and grow inside the phagocyte. These macrophages again lyse and die due to bacterial load [34]. Two to three weeks after infection, the third stage begins. T cell immunity develops, and lymphocytes drift to the region of infection. Presentation of mycobacterial antigens to the T cells causes their stimulation, resulting in the release of *γ*-interferon and other cytokines. The *γ*-interferon activates macrophages to secrete IL-12, TNF-*α*, IL-8, and other proinflammatory cytokines. Fast growth of the Mtb stops and, at this stage, the host cell develops cell-mediated immunity. Those that are outside of cells are resistant to antibody-activated complement attack due to the high lipid content of mycobacterial cell wall. Cell-mediated immunity is also responsible for much of the pathology of tuberculosis. Tissue damage can also take place when activated macrophages release lytic enzymes, reactive intermediates, and various cytokines. It is at this stage that the immune system, specifically the macrophages, will enclose the microorganisms inside tubercles. In between these structures, the atmosphere is anoxic and acidic and prevents the growth of mycobacteria. In-between of these structures is anoxic and acidic, preventing the growth of mycobacteria. This balance between host and mycobacterium is called latency which is one of the hallmarks of TB. In the fifth and final stage, the tubercles may dissolve by many factors such as malnutrition, immunosuppression, steroid use, or HIV infection. For unknown reasons, the centers of tubercles may liquefy, providing an outstanding growth medium for the microbe which now begins to grow rapidly in the extracellular fluid. The large number of bacteria and the immune response against them eventually cause the lung tissue near the tubercles to become necrotic and form a cavity [35]. Most tuberculosis infections stop at stage three.

It is an established fact that a cell-mediated immune response involves CD4+ (helper) and CD8+ (cytotoxic) T cells and both play significant role in protection against TB. Antibacterial activity of macrophages is enhanced by the CD4+ (helper) T cells by releasing cytokines like interferon-*γ* (IFN-*γ*) and TNF, whereas CD8+ cells destroy infected macrophages and possibly Mtb by releasing different cytotoxic mediators like perforins, granzymes, and granulysin [2]. In spite of our enhanced information of immune response to Mtb, the type of immune response required for the effective immunity that can be induced by vaccination is not fully understood [36].

## 7. Nanotechnology-Based Therapies

Over the past few years, the budding use of nanotechnology-based therapy has been researched for replacing the administration of antibiotics or other drugs in the free form with an access using drugs that are encapsulated with nanoparticle [13].

## 8. Nanoparticles and Tuberculosis

Nanobead delivery has a remarkable feature of slow, sustained, and controlled release from a biodegradable particle. Different animal models have been tried out to develop an antibiotic therapy based on polymer technology against* M. tuberculosis* but unfortunately not a single model stands up to the expectation or imitates all the features of human TB [31].

The sizes of nanoparticles which are defined as submicron (<1 um) colloidal particles are used as drug delivery vehicles. For therapeutic purposes, drugs can be covalently embedded to the particle surface or can be incorporated in the matrix of the particle [37]. Nanoparticles comprise biocompatible and biodegradable materials such as polymers, which can be either natural (e.g., gelatin and albumin), synthetic (e.g., polylactides and polyalkylcyanoacrylates), or solid lipids (SLN^R^ and NLC^R^) [38].

Nanoparticles are taken up more efficiently by cells than larger molecules which make them a promising transport and delivery system. These carriers are adapted to enable controlled, slow, and persistent drug release from the matrix [39]. Several methods have been discussed explicitly in several reviews for preparation and characterization of nanoparticles (Table 2).

Below are the advantages of nanoparticle-based drug delivery system for treatment of tuberculosis:

1. High constancy/longer time period
2. High carrier ability; that is, multiple drugs can be encapsulated in the matrix
3. Less side effects compared to conventional drugs
4. Increased bioavailability (slow, sustained, and controlled drug release)
5. Viability of various routes of administration like oral delivery and inhalation
6. Minimal side effects and improved compliance

## 9. Oral Delivery of ATD Nanomedicine

Commonly, uptake of nanoparticles occurs through various methods: (1) transcytosis through M-cells, (2) intracellular uptake and transport via the epithelial cells present in the intestinal mucosa, and (3) uptake by Peyer's patches. Oral administration is possible due to the stability and sustained release of drugs from nanoparticle. Pandey et al. (2004) [40] have verified the efficacy of anti-TB drugs after oral administration.

Three leading antitubercular drugs, namely, rifampin, isoniazid, and pyrazinamide, were brought into play for the tuberculosis treatment. These drugs were prepared by solvent evaporation method and by double emulsion process, which were encapsulated by PLG NPs [40]. Drug levels were maintained above the least inhibitory concentration (MIC90) in mice after a single oral administration of drug-loaded PLG NPs for 6 to 9 days in the plasma. Though free drugs were vacant from plasma within 12–24 hours following the oral administration [40], complete bacterial clearance from the organs was observed when Mtb infected mice were treated with the nanoparticle-bound drugs (5 oral doses every 10th day). Only after administration of 46 doses were free drugs able to generate same cause. It is worth stating that, when carried out in larger animal like Guinea pigs, similar findings were observed related to the pharmacokinetics, biodistribution, and chemotherapeutic effectiveness of the formulation [41]. However, in TB treatment, the major concerns which result into low compliance of patients to ATD treatment are dosing frequency and duration of chemotherapy. For enhancing patient's compliance during ATD treatment, WHO suggested the addition of the ethambutol to the intensive stage of chemotherapy as the drug is known to boost the rate of sputum conversion [42]. Hence, the chemotherapeutic prospective of PLG NPS-encapsulated EMB when administered with other three encapsulated front-line ATDs was evaluated. Subsequently, when a single oral therapeutic dose of drug-loaded NPs was administered to mice, therapeutic drug concentrations were retained in the plasma for 3 days, 6 days, and 8 days in the case of EMB, RIF, and INH-PZA, respectively [43]. On the other hand, free drugs were not spotted in the plasma after 12 hours of intravenous or oral administration, so ATD-loaded PLG nanoparticles were administrated to Mtb infected mice at every 10th day in comparison to free drugs administered daily. This yet again establishes the worth of nanomedicine [44].

## 10. Ligand-Conjugated Oral-ATD Nanomedicine

Polymeric nanoparticles act as bioadhesive in gastrointestinal tract. PLG nanomedicine was further improved by the addition of ligand better known as bioadhesive ligand. Lectins, a mucosal ligand, have been shown to improve the adhesion of nanoparticle to the mucosal surface which increases the absorption associated with drugs and its bioavailability [45]. Wheat germ agglutinin's receptors are distributed on intestinal and alveolar epithelium, thus making it useful for oral as well as aerosol drug delivery [46], and its covalent attachment to PLG has been shown to boost the efficacy of anti-TB drugs [20]. Upon oral/aerosol administration of wheat germ agglutinin-coated PLG nanoparticles in mice, it showed prolonged plasma levels of 6 to 7 days for RIF and 13 to 14 days for INH and PYZ as compared to uncoated PLG-NPs (4–6 days for RIF and 8-9 days for INH and PYZ). Three oral/nebulized doses of these lectin-coated nanoparticles every 14 days (versus 45 daily doses of free drugs) resulted in complete bacterial clearance. All three drugs were present in lungs, liver, and spleen for 15 days [20]. It also has extensive application in drug delivery due to its low immunogenicity [47].

The lasting circulation of drugs encapsulated in wheat germ agglutinin-grafted nanoparticles may be because of the fact that lectins improve prolonged adhesion of the particles to the intestinal shell to permit an increase in the time interval for absorption and also increase in the concentration gradient between serosal and luminal sides of the membrane [36].

## 11. Pulmonary Delivery of ATD Nanomedicine

Pulmonary TB is the most ubiquitous form of the disease, and the respiratory path represents a unique means of delivering ATDs directly to the lungs. Reduction of systemic toxicity and accomplishing higher drug concentration at the chief site of infection are the promising advantages of direct delivery of drug to the lungs. Inhalable NPs possess an enhanced ability of mucosal adherence, particle delivery, and net drug delivery to the lungs [18, 48].

## 12. Intravenous Delivery of ATD Nanomedicine

There are three injectable routes of drug delivery. Among these, intravenous administration of drugs results in immediate availability of all the drug molecules and increases bioavailability. Other routes like subcutaneous and intramuscular routes also provide similar bioavailability as compared to intravenous route [49].

A single subcutaneous injection of PLG nanoparticles loaded with RMP, INH, and PZA resulted in sustained therapeutic drug levels in plasma for 32 days and in lungs or spleen for 36 days. This produced complete sterilization of organs of Mtb infected mice and demonstrated better therapeutic efficacy as compared with daily oral free drugs (35 doses) [19]. This demonstrates the better efficacy of nanoparticles compared to microparticles. Microparticles with diameter of more than 1 *μ*m cannot be administered via intravascular route; however, nanoparticles are small enough to pass through [50].

Clofazimine is a new promising anti-TB drug for mycobacterial infection. However, use of this drug was limited because of its less solubility. To defeat this problem, a novel approach was applied. Clofazimine was formulated as a nanosuspension (particle size, 385 nm). Upon intravenous administration of this formulation, a significant reduction of colony-forming unit count in the liver, spleen, and lungs of mice infected with* M. avium* was observed [51]. The effects of the clofazimine nanocrystalline were found to be similar to the liposomal formulation used as a control in this study. This study clearly demonstrates the importance of nanotechnology to overcome the solubility problems and toxicity of drugs [52].

## 13. Liposome-Based Drug Delivery Systems

Liposomes are miniature closed vesicles consisting of phospholipid bilayer enfolding an aqueous section [53]. They have been broadly studied as a promising drug delivery model for bioactive compounds because of their sole ability to encapsulate both hydrophilic and hydrophobic drugs. To examine better chemotherapeutic efficacy in animal models like mice, liposomes have been evaluated for the constant delivery of anti-TB drugs [31, 54]. Few drugs like amphotericin B (fungal infection) and doxorubicin (breast cancer) have been approved for human use [55, 56].

When administered, phagocytic cells promptly recognize these carriers and vacant them from the blood stream. In order to avoid removal/clearance and broaden circulation times, liposomes are usually PEGylated. An established work discovered the incorporation of gentamicin into liposomes and evaluated the antimicrobial activity compared to that of the free drug in a mouse model of dispersed* M. avium* complex infection [57]. It was seen that the encapsulated drug considerably reduced the bacterial load in liver and spleen; however, sterilization was not found. Similar outcome was obtained with diverse liposome-entrapped second-line antibiotics [58–60].

Vigorous take-up by alveolar macrophages is effective against intracellular pathogens with two main advantages of using liposomes [61]. As liposomes are susceptible to intestinal lipases, they must be administered by either respiratory means or intravenous route. Nonspecific uptake by mononuclear phagocyte system (MPS) of liver and spleen can be reduced by the inclusion of PEG in the liposomal formulations [21, 62].

Upon administering Mtb infected mice twice a week for 6 weeks, it was observed that liposomes encapsulated drugs (rifampicin or isoniazid alone) were more powerful in clearing mycobacterial infection when compared to the free drugs. Dose was fruitfully reduced to one weekly administration for 6 weeks when these two front-line ATDs were coadministered in liposomes. As per histopathological examination, no hepatotoxicity was reported and was supported by levels of serum albumin, alanine aminotransferase, and alkaline phosphatase [63].

When INH and rifampin encapsulated in the lung specific stealth liposome were used against Mtb infection, it was revealed that liposome encapsulated drugs at and below therapeutic concentration were more effective than free drugs against TB [21].

## 14. Microemulsions as Potential Anti-TB Drug Delivery Systems

The concept of microemulsions was first introduced by Hoar and Schulman in 1943 [64]. Danielsson and Lindman [65] have correctly defined microemulsion as follows: “microemulsion is a system of water, oil and an amphiphile (surfactant and co-surfactant) which is a single optically isotropic and thermodynamically stable liquid solution.”

Microemulsions in recent years have gained a lot of attention for the development and design of new drug delivery systems because of their thermodynamic stability, high diffusion and absorption rates, ease of preparation, and high solubility [66, 67]. They aid in the improvement of drug bioavailability [68], resistance against enzymatic hydrolysis, and reduced toxicity [69]. The droplets of microemulsions are very small and they are thermodynamically stable. In stable microemulsions, the droplet diameter is usually within the range of 10–100 nm and hence these systems are also termed as nanoemulsions [70]. Microemulsions have wide applications in colloidal drug delivery systems for the purpose of drug targeting and controlled release. There are three different types of microemulsions depending on composition: (a) oil-in-water (o/w), (b) bicontinuous, and (c) water-in-oil (w/o) microemulsion [71].

Mehta et al. studied the use of tween-based microemulsion systems for potential application as a drug carrier for the anti-TB drug rifampicin [72]. They formulated microemulsion composed of oleic acid + phosphate buffer (PB) + Tween 80 + ethanol and examined its potential as a delivery system for an antitubercular drug. They studied numerous structural features with various physiochemical methods such as electron microscopy, NMR, optical microscopy, and dissolution and release kinetics and concluded that microemulsions containing Tween 80 were successful, since they encapsulated the anti-TB drugs (RIF, INH, and PZA) in different combinations by means of conductivity and viscosity, with no precipitation or phase separation [72]. In another study, Kumar performed the inclusion of INH in O/W microemulsion or W/O microemulsion comprising TX100 : AcOH (1 : 1), followed by cetyltrimethylammonium dichromate (CTADC), chloroform, and water; this microemulsion system presents the opportunity of sustained release, increasing drug solubility and bioavailability [73]. Talegaonkar et al. studied a means of concentrating RIF by microemulsion for oral drug delivery in order to make this drug more efficient, which was composed of a reaction product of a castor oil and ethylene oxide [74]. This study established that RIF was effective and may likely prevail over the problem, since lowering the dose lessens the toxicity.

## 15. Solid Lipid Nanoparticle-Based Anti-TB Drug Delivery System

Liposomal degradation by intestinal lipases forbids their use by oral route. The SLNs are a nanocrystalline nanosuspension in water and can be administered orally with many positive key points like extensive steadiness and better encapsulation effectiveness than liposomes and polymeric nanoparticles and the formation process also involves nominal amounts of organic solvents. In SLN, in order to produce lipid nanoparticles, the drug is mainly entrapped in solid lipid matrix [75]. The main hallmarks of SLNs are superior tolerability (due to their origin from physiological lipids), scaling up feasibility, the ability to incorporate hydrophobic or hydrophilic drugs, and an improved stability of incorporated drugs [76].

When compared to liposomes and polymeric nanoparticles, SLNs have longer/higher stability and enhanced encapsulation efficiency and involve minimal quantity of organic solvents, respectively. A single oral administration of ATD-loaded SLNs to mice resulted in drug identification in the plasma from 3 hrs and lasted up to 8th day [41]. At each time peak, plasma drug concentrations were at or above the least inhibitory concentration (MIC90), whereas free drugs were cleared from the circulation within 12 hrs of oral administration [77].

The chemotherapeutic prospective of the formulation was predicted via the respiratory route in Guinea pigs. It was seen that a prolonged drug release was preserved for 5 days in plasma and for 7 days in organs. Seven doses of the formulation resulted in complete clearance of bacilli in replacing 46 conventional doses [42]. Further, ATD-loaded SLN was also evaluated via oral course and better results were obtained as the drug level could be retained in plasma for 8 days and in organs for 9-10 days. In* M. tuberculosis* H37Rv infected mice, to achieve complete sterilization in the lungs/spleen, 5 oral doses at every 10th day of drug-loaded SLNs were sufficient, whereas free drug needed administration of 46 daily oral doses to get the same result. SLN-based antitubercular drug majorly reduced the dosing rate of recurrence and improved bioavailability [41].

## 16. Niosomes-Based Anti-TB Drug Delivery System

Niosomes are thermodynamically stable liposomes like colloidal particles formed by self-assembly of nonionic surfactants and hydrating mixture of cholesterol in aqueous medium resulting in multilamellar systems, unilamellar systems, and polyhedral structures [78, 79]. They are vesicular systems analogous to liposomes which can be used as carriers of amphiphilic and lipophilic drugs. Niosomes hold all the characteristics of liposomes except that they are composed of a surfactant bilayer with its hydrophilic ends exposed on the outside and inside of the vesicle to the aqueous phase, while hydrophobic chains face each other within the bilayer [80]. The bilayer system in niosomes is composed of uncharged single-chain nonionic surface-active agents, while double-chain phospholipids (neutral or charged) are seen in the liposomal structures. The sizes of niosomes lie in nanometric scale and are microscopic. The particle size ranges from 10 nm to 100 nm. Niosomes have drawn a lot of interest in the field of modern drug delivery systems due to their significant features such as biodegradability, biocompatibility, chemical stability, low production cost, easy storage and handling, and low toxicity [81].

Niosomes are one of the most promising carriers that can be used in targeted drug delivery systems through diverse principal schemes of drug targeting. The potential of niosomes to deliver drugs in a controlled/sustained manner in different applications and therapies has led to bioavailability development and continuous therapeutic effect over a longer phase of time [82].

Karki et al. formulated a niosomal drug delivery system of antitubercular agents such as isoniazid that has exceptional potential for development into a low dose performed with effective treatment for tuberculosis [83]. Niosomes can also be used as orally controlled release systems. For example, antitubercular hydrophilic drugs such as isoniazid and pyrazinamide which are orally active showed sustained drug release from the tyloxapol niosome membrane [84].

Rani et al. prepared niosomes of rifampicin and gatifloxacin by lipid hydration technique [85]. They studied the bactericidal activities of the niosomal formulation by the BACTEC radiometric technique using the resistant strain (RF 8554) and sensitive strain (H37Rv) of* Mycobacterium tuberculosis* which showed inhibition and reduced growth index. This means that rifampicin and gatifloxacin niosomes provided extensive release of drugs, which was optimum to provide a decreased dose, fewer days of treatment, and more patient compliance [85].

Thomas and Bagyalakshmi concluded that all three polymers such as Brij-35, Tween 80, and Span-80 used for the successful formulation of pyrazinamide niosomes helped avoid hepatotoxicity by keeping the cholesterol content constant. FTIR results showed that this drug and the polymer used were compatible. It was seen that Span-80 formulation had the highest percentage release when compared to other formulations [86].

## 17. Alginate-Based Anti-TB Drug Delivery System

Guluronic acid and mannuronic acid are the natural copolymer of alginic acid. For the supportive treatment of reflex esophagitis, US Food and Drug Administration authorized the oral use of alginate. Alginate is a naturally occurring biopolymer which has been getting louder application in various fields. It serves numerous purposes like disintegrating and binding agent in tablets, solidifying and suspending agent in water-miscible gels, creams, and lotion, and stabilizer for emulsions. It has various applications such as solidifying agent, gelling agent, and colloidal stabilizer in food and beverage industries. There are other diverse properties which make alginate an ultimate drug delivery model. These consist of (1) aqueous background within the matrix, (2) adhesive interface with intestinal epithelium, (3) drug encapsulation process without the use of organic solvent, (4) high gel porosity permitting high diffusion rate of macromolecules, (5) capacity to control this porosity with easy coating measures using polycations, (6) biodegradation of the system under physiological conditions, (7) sustained drug release, and (8) being nontoxic. For all the rationales mentioned above, alginate is utilized as a carrier for the controlled discharge of several molecules of clinical concern including indomethacin [87], gentamicin [88], insulin [89], anticancer drugs [90], and ATDs [91–93].

González-Rodríguez et al. have developed ATD-loaded alginate nanoparticles by means of ionotropic gelation with fairly high encapsulation efficiency ranging between 80 and 90% for rifampicin, 70 and 90% for isoniazid and pyrazinamide, and 88 and 95% for ethambutol [94]. After oral administration of this formulation, drugs were observed in plasma for 7, 9, 11, and 12 days for ETB, RIF, INH, and PYZ, respectively, and in tissues until day 15, in contrast to free drugs which were cleared from blood after 12 to 24 hrs and were detectable in tissues only until day 1 [94]. Oral administration of 3 dosages fortnightly in Mtb infected mice showed the complete clearance of bacilli from the organs as compared to 45 dosages of free drugs [95] which were comparable to the results obtained in Guinea pigs by the oral and the respiratory route [96, 97]. Therefore, these results advocate the supremacy of alginate NPs over other PLG NPs [43].

In another study, important observations were made when chitosan was added to the alginate. Alginate supplemented with chitosan resulted in improved pharmacokinetics and therapeutic efficacy and reduced the drug dose to half [98]. Another advantage of alginate NP is that it allows more loading of drugs with lower consumption of polymer.

However, there is some alarm/unease that the organic solvents used to suspend polymer may have detrimental side effects. On the basis of this, alginate, a water-soluble polymer which is free from organic solvent and may be preferred, has been used effectively against* M. tuberculosis* in the course of oral and aerosol administration in mice and Guinea pigs [43]. Being hydrophilic in character, alginate avoids swift clearance by the mononuclear phagocyte system on intravenous administration. This reports a long circulation half-life. This system shows obvious advantages over the established neutral polymers or liposomes.

Alginate NPs have been developed and stabilized with poly(L-lysine)-chitosan, encapsulating ATDs (RIF, INH, PZA, and EMB). The ratio for drug to polymer was kept at 7.5 : 1, which was considered to be better than PLG NPs, where the ratio was 1 : 1. Thus, the alginate formulation allocates more loading of the drug with minor consumption of the polymer. The formulation provides a persistent release for 7–11 days in plasma and 15 days in the organs following a single oral dose [99].

## 18. Conclusions and Future Perspective

In developing and underdeveloped countries, infectious diseases are foremost issues of health concern. Particularly in the developing countries, almost one-third of the destitute population does not have proper access to vital medicines. Tuberculosis has a very big impact on developing nations. In this scenario, for the effective control of the TB, drug-resistant TB acts as a major challenge. The aim is to find an answer to this by creating or producing better and more effective drugs that lessen the period of treatment, reduce drug toxicity, and have longer bioavailability. Till now, apart from few drugs like quinolones and rifamycins, no major contributions have been made to the ATD therapy and certainly an effective TB vaccine has also remained equally elusive. The goal is to find out a solution to eradicate the transmission of causative organism but this is difficult, multifarious, and thorny due to the difficulty of diagnosis, multidrug resistance, and patients' low compliance to treatment. A number of new anti-TB prospective drugs are in channel but the major drawbacks associated with these drugs include lack of rigorous research work, high cost, difficulty in aiming MDR and dormant bacilli, and drugs toxicity. These are the reasons which stimulate for the search of new and unique alternative therapeutic drug. It was observed that nanoparticle-based therapy could offer a potential advantage over conventional therapy for TB, which has a great potential to diminish the drug regimen and improve patient's compliance. The use of nanoparticles is one of the promising steps taken presently to improve vaccination against TB. Advancements in the nanoparticle-based delivery systems represent a commercial, practical, and most promising substitute for potential TB chemotherapy. The superior drug bioavailability and therapeutic usefulness are even at low therapeutic doses of the formulation and period of chemotherapy can also be reduced. All these aspects are vital in substantially curtaining the expenditure of treatment, reducing interactions with anti-HIV drugs, and improved management of MDR-TB and latent TB. The above facts imply the conclusion that nanoparticles have tremendous potential for the treatment of TB. Their foremost advantages such as long shelf life, dipping of dosing frequency, and enhancement of drug bioavailability make DOTS more convenient and reasonable. Oral and inhalation routes of drug administration are another reason why it makes nanoparticles more feasible. To conclude, the success of this technology will depend on the toxicological profiles linked with understanding the fate of polymeric components of nanocarriers in the body. Based on this opinion, drug carriers made from natural polymers like alginate and chitosan suggest a smart outlook but still they require more explorations. Appropriate clinical studies should be done in order to find out whether or not nanoparticle-based drug delivery system might be much anticipated solution for improving the patient compliance in TB chemotherapy.

## Figures and Tables

**Table 1:** TB drugs development in pipeline.

	Discovery	Preclinical development	Clinical development		
			Phase 1	Phase 2	Phase 3
Existing drugs redeveloped or repurposed for TB				Linezolid, rifapentine	Gatifloxacin, moxifloxacin

New drugs developed specifically for TB	Nitroimidazole, riminophenazines, translocase-1 inhibitor, InhA inhibitor, GyrB inhibitor, LueRS inhibitor, pyrazinamide analogues, diarylquinoline, spectinamides	CPZEN-45, SQ641, SQ609, DC159a, Q201	AZD5847	Bedaquiline (TMC-207), PA-824, SQ-109, PNU-100480	Delamanid (OPC67683)

**Table 2:** Therapeutic efficacy of nanoparticle-based antitubercular drug delivery systems.

Delivery system	Drug : carrier ratio	Use of organic solvents	Drug encapsulation efficiency	Mode of delivery	Animal model	Duration of sustained drug release (days)		Sterilizing effect in lungs and spleen	References
						Plasma	Organ		
Synthetic									
(i) PLG nanoparticles	1 : 1 for each drug	Dichloromethane	60–70%	Oral Aerosol Subcutaneous	Mice Guinea pigs Mice	6–8 6–8 32	9–11 9–11 36	5 doses every 10 d 5 doses every 10 d Single injection	[14, 15] [16] [17]
(ii) Lectin functionalized PLG nanoparticles	1 : 1 for each drug	Dichloromethane	60–70%	Oral Aerosol	Guinea pigs Guinea pigs	6–14 6–14	15 15	3 doses fortnightly 3 doses fortnightly	[14] [15]

Natural									
(i) Liposomes	RIF 0.22 : 1 INH 0.14 : 1	Chloroform, methanol	35–45% 8–12%	Intravenous Aerosol	Mice, Guinea pigs Guinea pigs	5–7 2	7 5	2 doses every week single dose every 5–7 days	[15, 18] [19]
(ii) Solid lipid nanoparticles	1 : 1 for each drug	Acetone, ethanol	40–50%	Oral Aerosol	Mice Guinea pigs	8 5	10 7	5 doses every 10 d 7 doses weekly	[20] [21]
(iii) Alginate nanoparticles	7.5 : 1 for each drug	No	70–80%	Oral Aerosol	Mice/Guinea pigs Guinea pigs	8–11 8–11	15 15	3 doses fortnightly 3 doses fortnightly	[22] [22]
